# Modeling and Simulation of Human Body Heat Transfer System Based on Air Space Values in 3D Clothing Model

**DOI:** 10.3390/ma14216675

**Published:** 2021-11-05

**Authors:** Sara Mosleh, Mulat Alubel Abtew, Pascal Bruniaux, Guillaume Tartare, Emil-Constantin Loghin, Ionut Dulgheriu

**Affiliations:** 1 ENSAIT-GEMTEX Laboratory, 2 Allée Louise et Victor Champier, Lille Université, 59056 Roubaix, France; sara.mosleh@ensait.fr (S.M.); pascal.bruniaux@ensait.fr (P.B.); guillaum.tartare@ensait.fr (G.T.); 2 Ethiopian Institute of Textile and Fashion Technology, Bahir Dar University, Bahir Dar 1037, Ethiopia; 3 Faculty of Textile, Leather and Industrial Management, ‘Gheorghe Asachi’ Technical University of Iasi, Dimitrie Mangeron Bd., 53, 700050 Iasi, Romania; eloghin@tuiasi.ro (E.-C.L.); ionut.dulgheriu@academic.tuiasi.ro (I.D.)

**Keywords:** thermal comfort, mathematical modeling, human body-clothing-environment interaction, ease allowance, clothing design

## Abstract

Comfort can be considered as subjective feeling, which could be affected by the external ambient, by the physical activity, and by clothing. Considering the human body heat transfer system, it mainly depends on various parameters including clothing materials, external and internal environment, etc. The purpose of the current paper is to study and establish a quantitative relationship between one of the clothing parameters, ease allowance (air gap values) and the heat transfer through the human body to clothing materials and then to the environment. The study considered clothing which is integrated with the 3D ease allowance from the anthropometric and morphological data. Such incorporating of the clothing’s 3D ease control was essential to properly manage the air space between the body and the proposed clothing thermal regulation model. In the context of thermal comfort, a clothing system consisting of the human body, an ease allowance under clothing, a layer of textile materials, and a peripheral layer adjacent to the textile material was used. For the complete system, the heat transfer from the skin to the environment, which is influenced by thermoregulation of the human body, air gap, tissue, and environmental conditions were also considered. To model and predict the heat transfer between the human body and the temperature of skin and clothes, a 3D adaptive garment which could be adjusted with ease allowance was used. In the paper, a thermoregulatory model was developed and proposed to predict the temperature and heat within clothing material, skin, and air space. Based on the result, in general the main difference in the temperature of clothing and skin from segment to segment is due to the uneven distribution of air layers under the clothing.

## 1. Introduction

Comfort can be considered as a subjective feeling, which depends on the external ambient, and by physical activity and clothing. Normally it can be categorized as physiological and psychological comfort. Psychological comfort is the comfort by which it is required of each person to wear specific clothes with colors and design features to make them feel good [1]. By contrast, physiological comfort could be considered to be impacted by thermal balance, such as the relationship between body heat production and losses [2]. Clothing thermal behavior is one of the main parameters that affect human comfort and it is a very complex behavior that many researchers have studied [1,3,4,5]. Many factors could affect the clothing thermal behavior including dry thermal insulation, air permeability, water vapor permeability, moisture adsorption, etc. [6,7,8]. A comfortable climate is usually defined as “the condition of mind that expresses satisfaction with the thermal environment” [9,10]. The experience of human climate is basically for the interaction between six different parameters including air temperature, mean radiant temperature, relative movements of air humidity air, level of activity, and clothing thermal properties.

In terms of thermal feeling in the face of changing environmental factors, human body modeling is a topic that researchers have considered for years in terms of applications in architecture [5], design of cooling and heating systems, aerospace industry [11], medical sciences [12,13], clothing [14,15,16,17], etc. In recent decades, many models have been proposed in thermal comfort modeling, each of which has capabilities and limitations [18]. Most existing models for predicting body temperature conditions are based on the body energy balance equation. These models divide the body into different parts and layers and evaluate the body’s thermal conditions by computing the energy balance equation and obtaining the temperature for each part and layer [19]. Among the different available models, one which helps to predict the thermal comfort of residents is the Fangar model [1], mentioned as the most famous steady model, and the Gage model [20] is the most well-known non-steady model. In addition, these two models use a relatively simple approach to assess body temperature conditions and, because of this simplicity, have been cited in the ISO [21] and Ashrae [9] standards. The air gaps, also known as ease allowances, entrapped between the skin and the clothing inner surface that not only express the designer’s intention but also facilitate body movement, regulate micro climate, influence the thermal feeling of the human body and the thermoregulatory response of the body [22]. Some researchers have critically investigated the influence of its distributions on the design and performance of clothing [23,24,25]. In thermal protective clothing, most of the research has focused on measuring temperature and the value of moisture in different air gaps and garments. For example, one researcher used a 3D body scanning technique to compute the layer ease allowance distribution between the different-sized protective clothes and the manikin used to estimate clothes’ thermal protective performance [22]. Another researcher showed that the thermal insulation of experimental shirts increased with ease allowance values but began to decrease due to natural convection when the ease allowance thickness was higher than one cm or the air gap volume was greater than 6000 cm^3^ [26]. In this study, a mathematical model is proposed to predict the effect of the air gap on the clothing temperature and heat loss of a clothed human body so that the suitability of the air gap in terms of comfort temperature can be optimized. The main aim of the current study is to study and establish a quantitative relationship between the clothing ease allowance value and the heat transfer through the human body to clothing materials and then to the environment, considering the integrated clothing with 3D ease allowance from the anthropometric and morphological data.

## 2. Materials and Methods

### 2.1. Multi-Node Thermoregulatory Model

**Figure 1:**
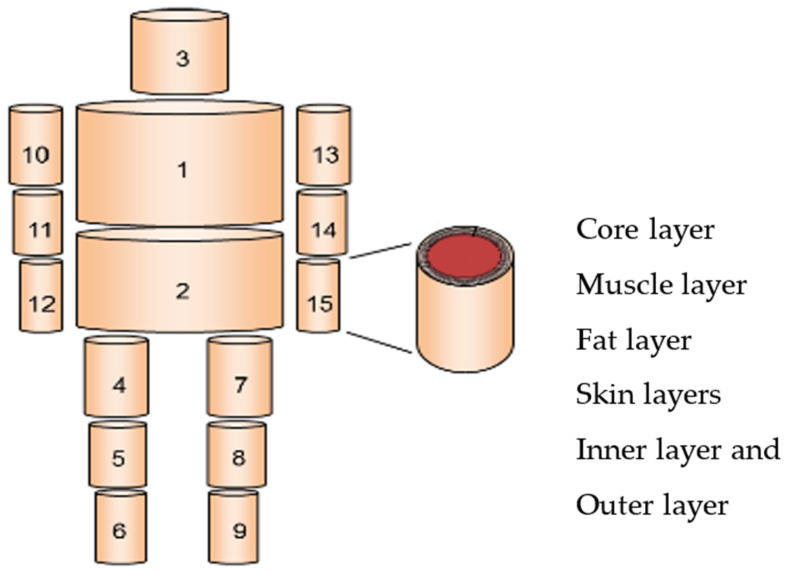
The different segmentation of 65 nodes model.

Human beings can adjust heat balance with the environment through thermoregulation and behavioral regulation. For example, human skin temperature changes dynamically according to human metabolic heat production, the air gap, clothing insulation, and environmental conditions, and influences the heat loss from a clothed human body. To simulate the effect of air gap thickness on the heat exchange between the human body and the environment, a mathematical model is developed to include human thermoregulatory and evaporative heat transfer from the skin to the environment through clothing. The starting point is Stolwijk’s model of thermoregulation [27], which has been developed to 65 nodes by sixteen segments, four layers, and central blood as shown in Figure 1. Another research study also evaluates the performance of the above; both Stolwijk and Tanabe model to predict the local skin temperatures of older people under thermal transient conditions [28].

In the present work, a model is proposed by developing the 65-node model by adding two more layers, namely the inner layer and outer layer of the clothing. This model has sixteen segments, which corresponds to the segmented thermal manikin. Moreover, every segment of the model is composed of six concentric layers such as core, muscle, fat, skin, inner and outer layer of clothing, and finally the central blood compartment. The heat balance equations for the different four layers and central blood compartment can be expressed through the following equation considering each layers’ and segment heat capacity C(i,j), heat production Q(i,j), heat transfer by blood flow to layers, heat transmitted by conduction D(i,j) between layers, heat loss by respiration RES(i,1) in core layer, evaporation heat loss at the skin surface E(i,4) and and B(i,j) is the heat exchange between each node and central blood compartment.

Core layer:

(1) $$C\left(i,1\right)\frac{\mathrm{dT}\left(i,1\right)}{\mathrm{dt}}=Q\left(i,1\right)-B\left(i,1\right)-D\left(i,1\right)-\mathrm{RES}\left(i,1\right)$$

Muscle layer:

(2) $$C\left(i,2\right)\frac{\mathrm{dT}\left(i,2\right)}{\mathrm{dt}}=Q\left(i,2\right)-B\left(i,2\right)+D\left(i,2\right)-D\left(i,2\right)\;$$

Fat Layer:

(3) $$C\left(i,3\right)\frac{\mathrm{dT}\left(i,3\right)}{\mathrm{dt}}=Q\left(i,3\right)-B\left(i,3\right)+D\left(i,3\right)-D\left(i,3\right)\;$$

Skin layer:

(4) $$C\left(i,4\right)\frac{\mathrm{dT}\left(i,4\right)}{\mathrm{dt}}=Q\left(i,4\right)-B\left(i,4\right)+D\left(i,4\right)-Q_{t}\left(i,4\right)-E\left(i,4\right)-Q_{\mathrm{nc}}\left(i\right)$$

Central blood:

(5) $$C\left(\mathrm{central}\;\mathrm{blood}\right)\;\frac{\mathrm{dT}\left(\mathrm{central}\;\mathrm{blood}\right)}{\mathrm{dt}}=\overset{16}{\underset{i=1}{\sum }}\;\overset{4}{\underset{j=1}{\sum }}\;B\left(i,j\right)\;$$

where, j(1 to 6) represents the six concentric layers.

### 2.2. The Multi-Node Thermoregulatory Model with Clothing

**Figure 2:**
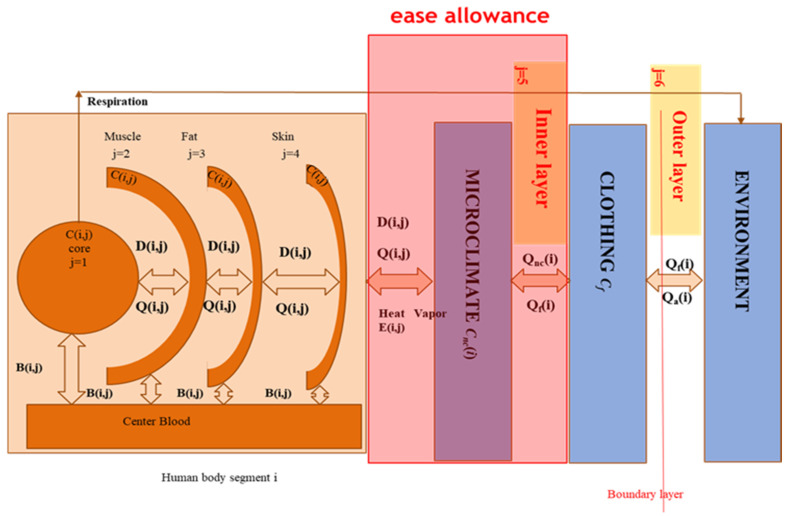
Human body-clothing-environment interaction system.

As discussed in the above section, the multi node model is developed by adding two (2) more layers, namely the inner layer and outer layer of clothing. This model also considers different kinds of heat transfer which usually occur inside and outside of the human body due to evaporation of sweating, respiration, and blood flow. In this case, we are going to see how the air gap (the ease allowance) could influence this rate of heat transfer. Based on the energy conservation law system as shown in Figure 2, it is also possible to establish the heat balance equations for inner and outer layers of clothing as follows;

Inner layer:

(6) $$A_{n}\left(i\right)L_{\mathrm{nc}}\left(i\right)C_{\mathrm{nc}}\left(i\right)\frac{\mathrm{dT}\left(i,5\right)}{\mathrm{dt}}=Q_{\mathrm{nc}}\left(i\right)-Q_{f}\left(i\right)$$

Outer layer:

(7) $$A_{\mathrm{cl}}\left(i\right)M_{f}\left(i\right)C_{f}\left(i\right)\frac{\mathrm{dT}\left(i,6\right)}{\mathrm{dt}}=Q_{f}\left(i\right)-Q_{a}\left(i\right)$$

where,

i(1 to 16) represents the sixteen segments of the body,
Qnc(i) is the direct heat transfer from the skin to the inner layer of the fabric for a clothed body segment,
Qf(i) is the direct heat transfer from the inner layer to the outer layer of the fabric,
Qa(i) is the direct heat flux transfer from the outer fabric layer to the environment,
An(i), Acl(i) are the surface area of the body and clothing, respectively,
Lnc(i) is the air gap thickness of segment I,
Cnc(i) is the volume heat capacity of air,
Cf is the specific heat of the fabric, and Mf is the mass of the fabric.

### 2.3. The Evaporative Heat Loss at the Surface of the Skin

The loss of heat from the surface of the skin could happen mainly through conduction of heat from the skin to the layer of still air around the body. It could also happen by convection of heat to the free air layers, and radiation from the skin, and evaporation of water. The evaporative heat loss also transpires through the skin and the respiratory system. The evaporation is responsible for around 10% to 25% of heat loss under thermal neutrality conditions. Moreover, the physical factors which direct the evaporative heat loss consist of the ambient air relative humidity, airflow velocity, and lung minute ventilation [29].

The E(i,4) is evaporative heat loss at the skin surface and could be given as:

(8) $$E\left(i,4\right)=E_{b}\left(i,4\right)+E_{\mathrm{sw}}\left(1,4\right)$$

Eb(i,4) is the water vapor diffusion heat loss through the skin. The skin diffusion is assumed to be 6% of Emax(i), and expressed as shown in the following equation:

(9) $$E_{b}\left(i,4\right)=0.061-E_{\mathrm{sw}}\left(i,4\right)E_{\max}\left(i\right)$$

where, E_max_(i) is maximum evaporative heat loss and is described as shown by Equation (10).

In the Stolwijk model, E_b_(i,4) values were given as constants which consists about 3–4% of E_max_(i), and the E_sw_(i,4) is the heat loss by evaporation of sweat.

(10) $$E_{\max}=h_{e}\left(i\right)\left(p_{\mathrm{sk},s}\left(i\right)-p_{a}\left(i\right)A_{n}\right)$$

where,

p_sk,s_(i) is the saturated vapor pressure on the skin surface,
p_a_(i) the ambient vapor pressure and
h_e_(i) is the evaporative heat transfer coefficient from the skin surface to the environment, expressed as a function of clothing vapor permeation efficiency I_cl_(i) by:
  (11) $$h_{e}={\mathrm{LRi}}_{\mathrm{cl}}\left(i\right)0.155I_{\mathrm{cl}}\left(i\right)+\left(\frac{i_{\mathrm{cl}}\left(i\right)}{h_{c}\left(i\right)f_{\mathrm{cl}}}\right)$$
  where,
  Icl(i) is the clothing thermal insulation factor for the individual segment,
  hc(i) is the convective heat transfer coefficient, and
  LR is the Lewis ratio.

The described thermal insulation system plays a great part in the determination of the clothing’s thermal comfort as well as the human thermal stress. According to the different existing studies, the wind speed and its direction, the movements of human body, the body posture and sweating, and the different physical parameters of clothing such as fabric properties (thickness, weight, density, and air permeability), clothing area factor and clothing design features (covering the area, clothing combinations, wearing style, air gap size and distribution, and air volume) have greatly influenced the clothing’s thermal insulation systems [30,31,32,33]. Besides, the air layer under the clothing also shows a much greater effect on clothing insulation as compared to the basic fabric properties including thickness and thermal conductivity [34]. The fit of the clothing with the body has also a greater impact on the clothing’s thermal insulation [35]. In general, the more the clothing fit becomes loose, the higher the thermal insulation will be realized than in tight-fitting clothing [36].

The clothing area factor, f_cl_(i) also plays a great role in the thermal insulation system. The devising procedure of such parameters was used in the literature [37] and utilizes clothed and nude manikin photograph analysis. The different images were taken at an angle of 0° and 90°. Later the clothed or the nude manikin surface areas were determined by analyzing the projection of the areas of the surface using Adobe Photoshop software.

The value for f_cl_ was then calculated as the ratio between clothed and nude areas as:

(12) $$f_{\mathrm{cl}}\left(i\right)=\frac{A_{\mathrm{cl}}\left(i\right)}{A_{n}\left(i\right)}$$

Based on the work in different laboratories, the thermal insulation of the nude manikin was determined based on the value of the surface air insulation thermal resistance (Ia). The dry heat insulation I_T_ $\left(\frac{\mathrm{square}\;\mathrm{meter}\;.\mathrm{Kelvin}\;}{\mathrm{watt}}=m^{2}\cdot K\cdot W^{-1}\right)$ was determined as follows for the dry manikins (Loughborough and Lund Universities) [38].

(13) $$I_{T}=\mathrm{Dry}-\mathrm{heat}\;\mathrm{insulation}\;\left(\mathrm{resistance}\right)=\frac{T_{\mathrm{skin}}-T_{\mathrm{ambient}}}{\mathrm{Dry}\;\mathrm{heat}\;\mathrm{loss}}$$

Besides, the intrinsic clothing insulation I_cl_ can be determined as:

(14) $$I_{\mathrm{cl}}=I_{T}-\frac{I_{a}}{f_{\mathrm{cl}}}\;(m^{2}\cdot K\cdot W^{-1})$$

The conversion from SI units to C_lo_ units can be performed by:

(15) $$I\left(\mathrm{clo}\right)=\frac{I\left(\mathrm{SI}\;\mathrm{units}\right)}{0.115}$$

### 2.4. The Sensible Heat Exchange at the Surface of Skin

The role of cloth in decreasing the transfer of heat among the different segments of body skin and environment changes from person to person and the type of clothing worn. Besides, the sensible heat exchange from the skin surface at temperature average is normally achieved by both conduction through clothing during wear, and convection and radiation from the outer clothing or skin surface to the surrounding medium [39]. The convective and radiant heat transfer coefficients were derived from the thermal manikin experiment [40].

The sensible heat exchange at the skin surface is then given by:

(16) $$Q_{t}\left(i,4\right)=h_{t}\left(i\right)\left(T\left(i,4\right)-t_{o}\left(i\right)\right)A_{n}\left(i\right)$$

where,

Q_t_(i,4) is the convective and radiant heat exchange rate between the skin surface and the environment.

t_o_(i) is the operative temperature and,

h_t_(i) is the total heat transfer coefficient from the skin surface to the environment is expressed by:

(17) $$h_{t}\left(i\right)=0.155I_{\mathrm{cl}}\left(i\right)+\left(h_{c}\left(i\right)+h_{r}\left(i\right)\right)f_{\mathrm{cl}}\left(i\right)$$

where, h_r_(i) is the radiant heat transfer coefficient.

### 2.5. The Sensible Heat Loss from the Skin

The heat loss of the skin to the environment $Q_{\mathrm{nc}}\left(i\right)$ is different for clothed and unclothed parts of the body segments. In the part of the body covered by clothing, the transfer of heat from the skin is through the ease allowance, the layers of the fabric, and the outer surface air layer of the clothing. In the unclothed body parts, on the other hand, heat transfer occurs through the boundary air layer of the bare skin and is released directly to the environment by natural convection and radiation. Heat transfer from the skin to the inner layer of clothing occurs by conduction or convection through the air gap layer and radiation between the skin and the inner surface of the clothing. According to the theory of Catton [41], heat transfer for air in a vertical enclosure of thickness δ occurs by conduction when R_a_ is less than 1000; if the value is greater, natural convection occurs. The Nusselt number ${\mathrm{Nu}}_{\delta }$ can be expressed as follows:

(18) $${\mathrm{Nu}}_{\delta }=\{\begin{array}{cc} 1 & \mathrm{Ra}\leq 10^{3}\\ 0.22{\left(\frac{\mathrm{Pr}}{0.2+\mathrm{Pr}}\mathrm{Ra}\right)}^{0.28}{\left(\frac{H}{\delta }\right)}^{-\frac{1}{4}} & 10^{3}\leq \mathrm{Ra}\leq 10^{10} \end{array}$$

Then, the heat transfer coefficient through the air gap $h_{\mathrm{nc}}\left(i\right)$ can be deduced as,

(19) $$h_{\mathrm{nc}}\left(i\right)={\mathrm{Nu}}_{\delta }\;\frac{k_{a}}{L_{\mathrm{nc}\left(i\right)}}=0.22{\left(\frac{\mathrm{Pr}}{0.2+\mathrm{Pr}}\;\frac{g\beta \left(T\left(i,4\right)-T\left(i,5\right)\right)H{\left(i\right)}^{3}}{\alpha v}\right)}^{0.28}{\left(\frac{H\left(i\right)}{L_{\mathrm{nc}}}\left(i\right)\right)}^{-1/4}\frac{k_{a}}{L_{\mathrm{nc}}\left(i\right)}$$

where,

Ra is the Rayleigh number,
Pr is the Prandtl number, H(i) is the height of the body segment I,
ka is the thermal conductivity of air,
g is the gravitational acceleration,
β is the thermal coefficient of volume expansion,
α is the thermal diffusivity, and υ is the kinematic viscosity.

Then the total sensible heat transfer from the skin to the inner layer of clothing can be expressed as:

(20) $$Q_{\mathrm{nc}}\left(i\right)={\;A}_{n}\left(i\right)\left\{h_{\mathrm{nc}}\left(i\right)\left(T\left(i,4\right)-T\left(i,5\right)\right)+\frac{\sigma \left(T^{4}\left(i,4\right)-T^{4}\left(i,5\right)\right)}{\left(\frac{1}{e_{n}}+\frac{1}{e_{f}}\right)-1}\right\}$$

where,

σ is the Stefan–Boltzman constant,
e_n_ is the emissivity of skin, and
e_f_ is the emissivity of the fabric.

The first term on the right side represents the thermal conduction or natural convection through the ease allowance (air gap) and the second term could denote the radiant heat transfer between the skin and inner layer of clothing.

Influenced by the intrinsic thermal insulation of fabric R_f_, the dry heat transfer through fabric could be also determined by:

(21) $$Q_{f}\left(i\right)=A_{\mathrm{cl}}\left(i\right)\frac{T\left(i,5\right)-T\left(i,6\right)}{R_{f}}$$

The dry heat transfer from the outer layer of fabric to the environment is composed of convective and radiant heat:

(22) $$Q_{a}\left(i\right)=A_{\mathrm{cl}}\left(i\right)h_{a}\left(i\right)(T\left(i,6\right)-T_{a}+{\sigma e}_{f}\left(T^{4}\left(i,6\right)-T^{4}\right)$$

where the first term represents natural convection from the outer layer of clothing to the environment, the second term-radiant heat transfer, and h_a_(i) is the natural convective heat transfer coefficient, which can be calculated using Kyunghoon’s theory [42] as:

(23) $$\mathrm{Nu}=\frac{h_{a}\left(i\right)H\left(i\right)}{k_{a}}=0.518{\left(\frac{c_{p}\rho^{2}g\beta \left(T\left(i,6\right)-T_{a}\right)H^{3}\left(i\right)}{k_{a}\mu }\right)}^{1/4}$$

where,

(24) $$h_{a}\left(i\right)=0.518{\left(\frac{c_{p}\rho^{2}g\beta \left(T\left(i,6\right)-T_{a}\right)H^{3}\left(i\right)}{k_{a}\mu }\right)}^{1/4}$$

C_p_ is the heat capacity at constant pressure,
ρ the density, and
µ is the viscosity.

Based on the energy conservation law, it is also possible to establish the heat balance equations for the clothing’s inner layer as follows.

## 3. Results and Discussion

### 3.1. The 3D Virtual Modeling of Cloth with 3D Ease Allowance

**Figure 3:**
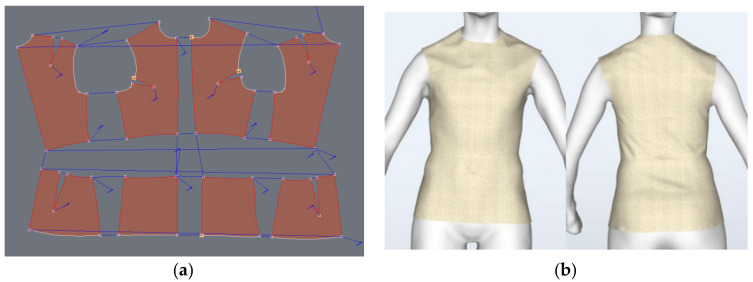
(**a**) 2D pattern from Design concept, (**b**) Garment [sewn] on 3D virtual model.

Using the Graphic model of the clothing, the ease allowance gap for the different clothing fits was determined. This allows us to adjust the 3D ease allowance for different clothing zones. In this study, a mathematical model is used to predict and optimize the effect of the air gap (ease allowance) on clothing temperature and heat loss of a clothed human body. Figure 3a shows the 2D pattern of the garment derived from the flattening of the 3D garment model, which can change with different fits. Figure 3b also shows the virtual 3D try on sample of the garment in different fits, where the garment fits closely to the body.

**Figure 4:**
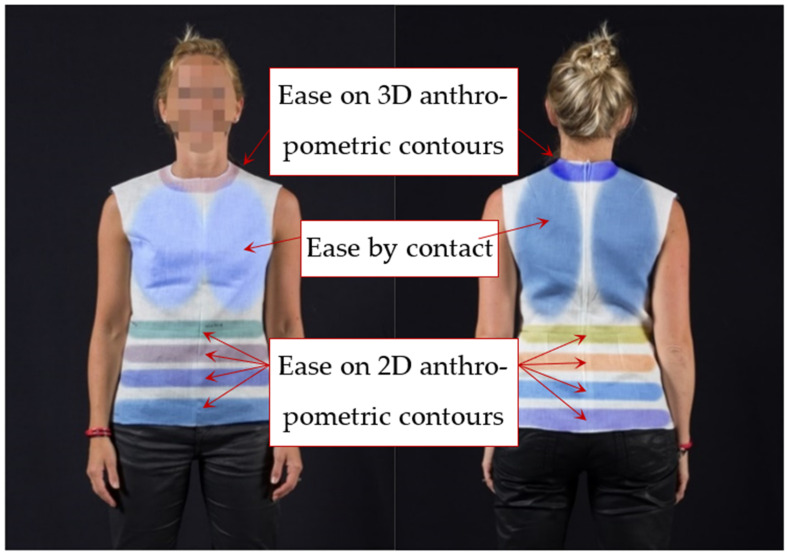
Ease allowance managed by zone.

In order to see the effect of the ease allowance gap, five different kinds of clothing fits for an upper garment (shirt) were used as shown in Table 1 (A, B, C, D, E). All the upper garments have the same pattern design and are made of the similar fabric materials (cotton plain fabric) [42]. However, in order to determine the effect of the air gap under the clothing, the proposed garments were designed with different values of 3D ease allowance. The 3D allowances were measured at different positions using the 3D design software, called Design concept of Lectra. The ease allowance, which was defined in the graphical model of the garment, can be considered as a 2D/3D ease allowance per zone as shown in Figure 4. Normally, two different techniques of ease allowance management are proposed, namely by contact and by anthropometric curves [43]. The ease allowance by contact appears in a level of the chest and scapula. It is a 3D ease allowance which is widely distributed on these two zones. The 2D ease allowance by anthropometric curves is defined in the contour plane concerned. However, the ease allowance measured at the level of the neck becomes 3D. Table 1 shows the five garments with different air gap thickness measurements. Basically, it is revealed that the values of the air gap thickness increase as the garment size increases.

By solving the heat balance equations of the human body and clothing, the effects of the air gap on the heat transfer of the human body heat transfer can be predicted, as well as the interaction between the human, clothing, and the environment. This can be achieved by comparing with the comfort standard and predicting at what value the air gap can provide comfort for the human body.

### 3.2. The Influences of Air Gap Thickness (Ease Allowance) on the Heat Transfer

**Figure 5:**
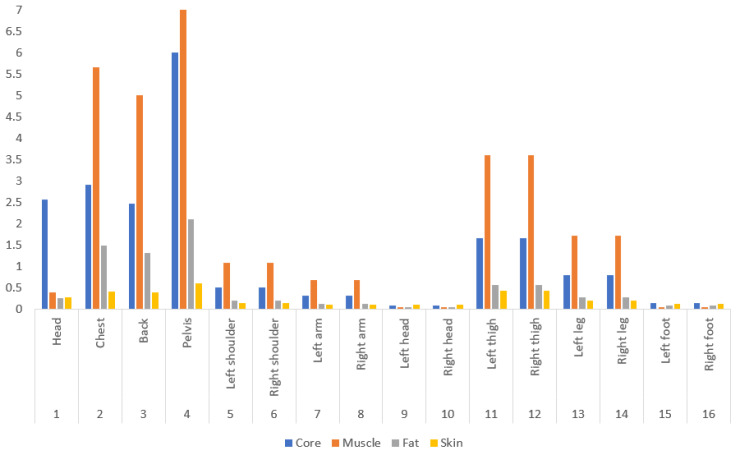
C(i,j) (Wh/°C) for central blood = 2.610.

The heat balance equations of the human body and clothing, as shown in Equations (1)–(7), were determined considering the specification of fabric (Table 2), heat capacity (Figure 5), the surface area of the body and clothing (Table 3), the height of the body segment (Table 4) and set-point temperature (Table 5) (which plays a role of “control target temperature”).

In our current study, the effects of the air gap on human body heat transfer, as well as the interaction between the human, clothing and the environment is predicted considering that the body is in the comfort temperature zone. The major parameters of the mathematical model are the air gap thickness, kind of fabric, environmental temperature and humidity, and metabolic heat production.

The human body at the standing position has its own values for the different parameters. For example, the metabolic heat production is around 1.7 Met (100.4 W/m^2^) [44], the velocity of wind at the skin surface, v is around 0.25 m/s, and the ambient and the radiant temperature is 25 °C and 20 °C, respectively. The heat loss from a clothed human body under the reference conditions based on the different measured values of 3D ease allowance of the five garments measured is shown in Table 3.

**Figure 6:**
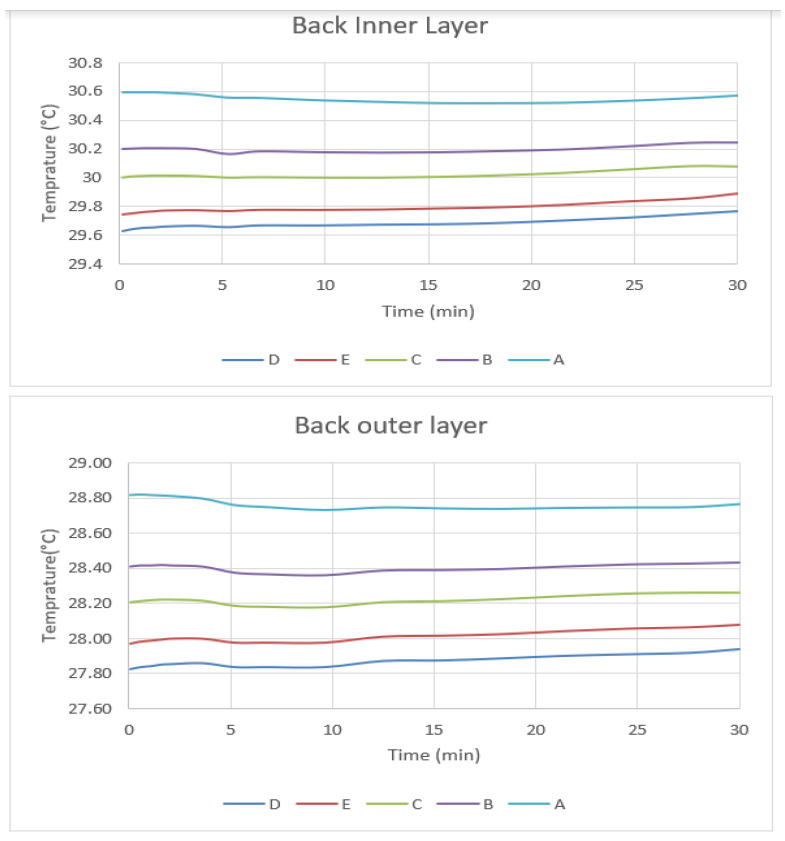
Inner layer and Outer layer temperature of five garment sizes.

**Figure 7:**
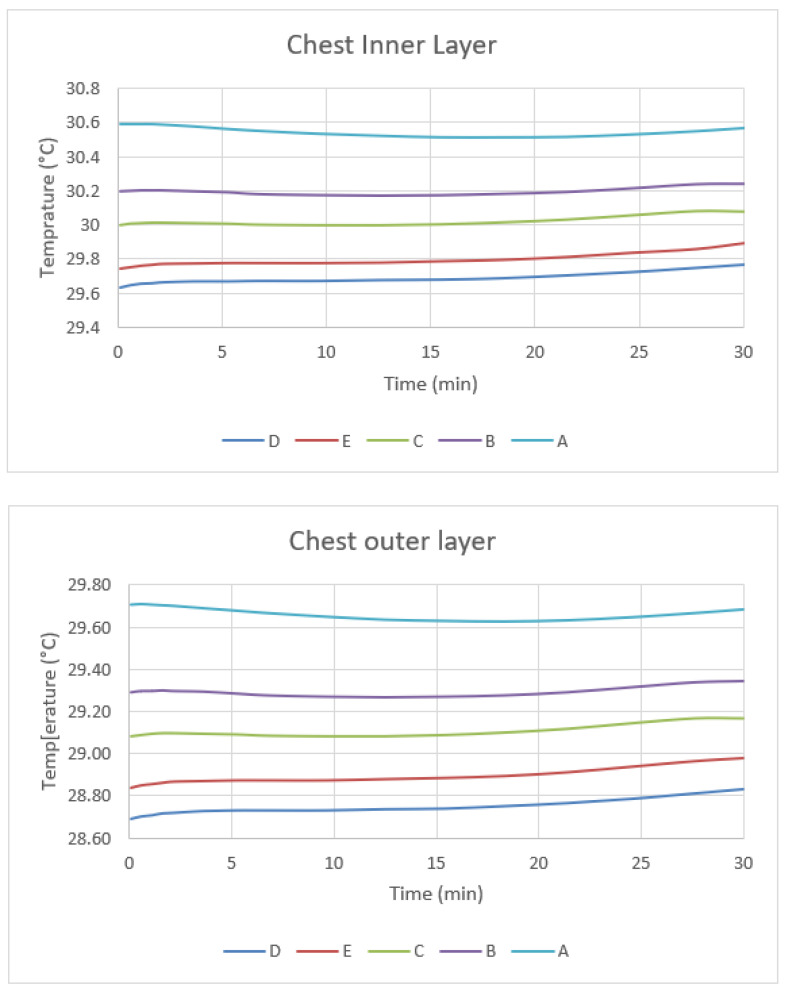
Inner layer and Outer layer temperature of five garment sizes in chest segments.

The apparent clothing temperatures at the chest and the back of the body after a thirty-minute simulation are shown in Figure 6 and Figure 7. The temperature at the inner and outer garment surfaces decreases with the thickness of air gap when the garment size is smaller than 1.55 cm. In this case, the air gap behaves as an insulating material that prevents heat transfer from the skin to the surface of the garment. However, the temperature increases when the garment size reaches garment category E with an air gap thickness of 1.95 cm. From the simulated results, natural convection starts when the thickness of the air gap is greater than 15 mm.

The onset of natural convection increases the heat transfer from the skin to the inner layer of the garment, which increases the apparent temperature of garment E. In the back segment, the only difference between back and chest due to lower parentage of fat and muscles is that the skin and garment temperature is lower than on the chest (in internal temperature, without activity), but the change behavior is the same as shown in Figure 6.

**Figure 8:**
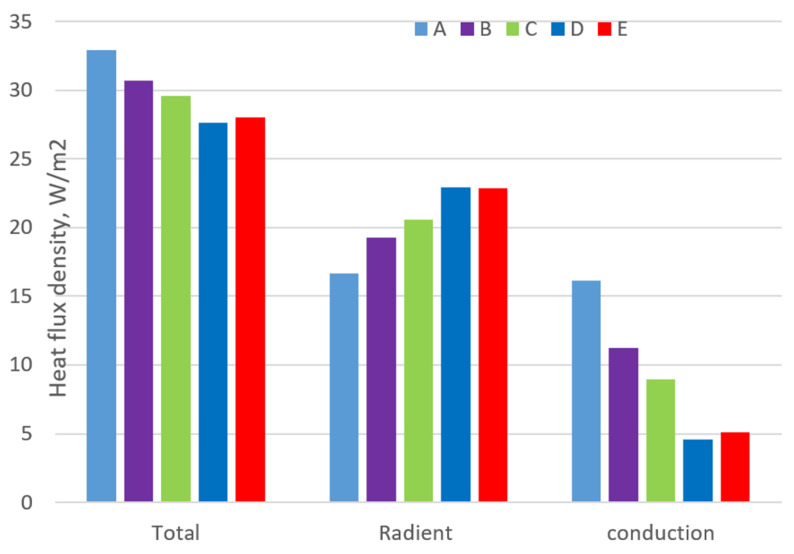
Dry heat loss of the different sizes of garments.

Besides, the air gap thickness effect on sensible heat loss through the air gap is shown in Figure 8. The total dry heat flux densities from skin decrease until the garment size is larger than D, among which the conductive component decreases from 50% to 18% of the total sensible heat flux densities. In comparison, the radiant heat increases from 55% to 88%. Since the radiation is independent of the air gap thickness, the radiant heat loss through the air gap increases due to an increase in the temperature difference between the skin and clothing surface. A large rate of reduction in the conductive component leads to a decrease in the total heat gain.

The total and conductive heat flux of garment E increases due to the result of the natural convection, but consequently its radiant component has decreased. This is because the air gap thickness increases linearly with the garment size, from tight to loose, whereas heat transfer from the human body to the environment occurs irregularly. The air gap undergarment D can efficiently block heat loss from the human body, creating the lowest apparent temperature of clothing.

## 4. Conclusions and Future Outlook

The air gap (ease allowance) between the body and clothing affects the heat loss from the skin to the environment. The current research paper describes the effects of the various air gaps on the heat transfer of a clothed human body. In the paper, a thermoregulatory model was developed and proposed to predict the temperature and heat for different clothing sizes. The result shows that the main difference in the temperature of clothing and skin from segment to segment is due to the uneven distribution of air layers under the clothing. Thanks to the adaptive 3D clothing that can adapt to the different air gaps (ease allowance of clothing), this model can predict the heat transfer between the human body and the temperature of the skin and clothing (inner and outer layers). The total dry heat flux densities from skin decrease until the garment size is larger than 1.55 cm, the conductive component reduces from 50% to 18% of the total sensible heat flux densities. The radiant heat increases from 55% to 88%. As far as the radiation is independent of the ease allowance, the radiant heat loss through the ease allowance increases because of the increasing temperature difference between the skin and clothing surface. Although the heat transfer from the human body to the environment is irregular, it was possible to propose a suitable air gap for different ambient temperatures. Thus, we have plans in our future study to validate our proposed model through experimental tests with the thermal manikin and optimize the size of the clothing based on different materials.

## Figures and Tables

**Table 1:** Average air gap (ease allowance) size.

Garment Code	A	B	C	D	E
Air gap thickness, cm	0.5	0.8	1.15	1.55	1.95

**Table 2:** Fabric specifications.

Fiber Type	Cotton
Area density, g/m^2^	126
Thickness, mm	0.312
Warp density, 1/10 cm	585.6
Weft density, 1/10 cm	284
Thermal resistant, clo	0.155

**Table 3:** An(i) and Acl(i).

i	Segment (i)	A_n_(i) (m^2^)	A_cl_(i) (m^2^)
1	Head	0.140	0.1400
2	Chest	0.175	0.1886
3	Back	0.161	0.1735
4	Pelvis	0.221	0.2347
5	Left shoulder	0.096	0.1020
6	Right shoulder	0.096	0.1020
7	Left arm	0.063	0.0669
8	Right arm	0.063	0.0669
9	Left head	0.050	0.0500
10	Right head	0.050	0.0500
11	Left thigh	0.209	0.2220
12	Right thigh	0.209	0.2220
13	Left leg	0.112	0.1189
14	Right leg	0.112	0.1189
15	Left foot	0.056	0.0560
16	Right foot	0.056	0.0560

**Table 4:** H(i) Height of the body segment.

i	Segment (i)	H(i) (m)
1	Head	0.1894
2	Chest	0.5504
3	Back	0.5023
4	Pelvis	0.2134
5	L-shoulder	0.1750
6	R-shoulder	0.1750
7	L-arm	0.4735
8	R-arm	0.4735
9	L-hand	0.1950
10	R-hand	0.1950
11	L-thigh	0.4490
12	R-thigh	0.4490
13	L-leg	0.4280
14	R-leg	0.4280
15	L-foot	0.2565
16	R-foot	0.2565

**Table 5:** Tset(i,j) (W/°C), Central blood temperature = 36.7 °C.

*i*	Segment	Core	Muscle	Fat	Skin	Inner Surface Fabric (A)	Outer Surface Fabric (A)
1	Head	36.9	36.1	35.8	35.6	35.6	35.6
2	Chest	36.5	36.2	34.5	33.6	31.5	29.2
3	Back	36.5	35.8	34.4	33.2	31.5	29.2
4	Pelvis	36.3	35.6	34.5	33.4	31.4	29
5	L-Shoulder	35.8	34.6	33.8	33.4	31.4	29
6	R-Shoulder	35.8	34.6	33.8	33.4	31.4	29
7	L-arm	35.5	34.8	34.7	34.6	32.5	30.5
8	R-arm	35.5	34.8	34.7	34.6	32.5	30.5
9	L-hand	35.4	35.3	35.3	35.2	35.2	35.2
10	R-hand	35.4	35.3	35.3	35.2	35.2	35.2
11	L-thigh	35.8	35.2	34.4	33.8	31.8	29
2	R-thigh	35.8	35.2	34.4	33.8	31.8	29
13	L-leg	35.6	34.4	33.9	33.4	31.6	29.3
14	R-leg	35.6	34.4	33.9	33.4	31.6	29.3
15	L-foot	35.1	34.9	34.4	33.9	33.9	33.9
16	R-foot	35.1	36.7	34.4	33.9	33.9	33.9

## Data Availability

The data presented in this study are available on request from the corresponding authors.
